# Microorganisms and Their Sensitivity Pattern in Septic Arthritis of North Indian Children: A Prospective Study from Tertiary Care Level Hospital

**DOI:** 10.1155/2013/583013

**Published:** 2013-10-22

**Authors:** Sanjay Yadav, Mandeep Singh Dhillon, Sameer Aggrawal, Sujit Kumar Tripathy

**Affiliations:** ^1^Department of Orthopaedics, Nehru Hospital, Post Graduate Institute of Medical Education and Research, Chandigarh 160012, India; ^2^Department of Orthopaedics, Room No. 247, Ayurvigyan Nagar, All India Institute of Medical Sciences, New Delhi 110029, India

## Abstract

*Background*. Septic arthritis is a true orthopaedic emergency. Important factors determining outcome are rapid diagnosis and timely intervention. Changing trends in microbiological spectrum and emerging drug resistance poses big challenge. Present study evaluates bacterial strains and their sensitivity pattern in septic arthritis of North Indian children. *Methods*. Fifty children with septic arthritis of any joint were evaluated. Joint was aspirated and 2 cc of aspirated fluid was sent for gram stain and culture. Blood cultures were also sent for bacteriological evaluation. *Results*. Fifty percent cases had definite radiological evidence of septic arthritis whereas ultrasound revealed fluid in 98% cases. Aspirated fluid showed isolates in 72% cases. The most common organism was *Staphylococcus aureus* (62%) followed by *Streptococcus pneumoniae* and Gr. B *Streptococcus*. Blood culture could grow the organism in 34% cases only. The bacterial strain showed significant resistance to common antibiotic cocktail in routine practice. Resistance to cloxacillin and ceftriaxone was 62% and 14% respectively. No organisms were resistant to vancomycin and linezolid. *Conclusion*. *S. aureus* is still the most common organism in septic arthritis. Though a significant resistance to common antibiotic cocktail is noticed, the strain is susceptible to higher antibiotics. We recommend using these antibiotics as an empirical therapy till culture and sensitivity report is available.

## 1. Introduction

**Figure 1:**
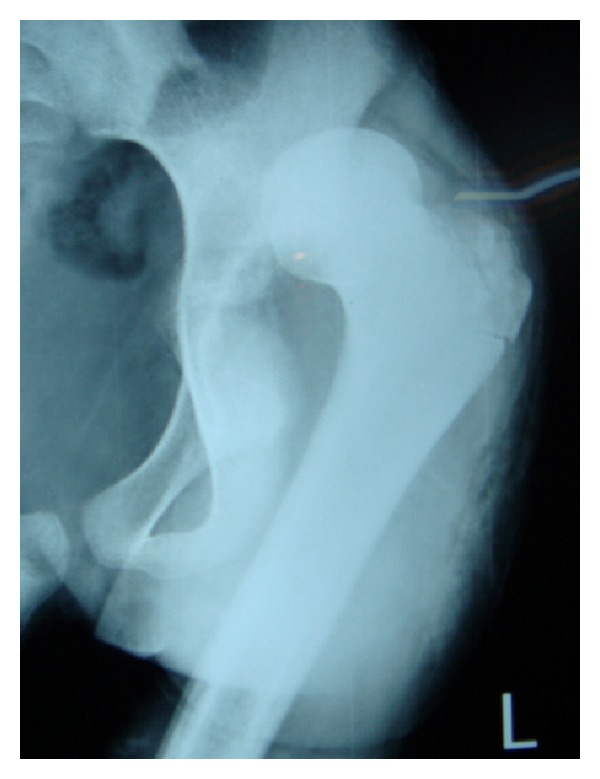
Septic arthritis causing pathological dislocation.

Septic arthritis is a true orthopaedic emergency. Delay in its diagnosis and treatment can lead to disastrous complications like destruction of articular cartilage, physeal damage, and dislocation of joints (Figure 1) [1–3]. Despite significant improvement in medicine with availability of better antibiotics, septic arthritis is still a major cause of morbidity. The cause is multifactorial as there is a shift in the microbiological spectra and epidemiology with emerging antibiotic resistance. This also has a distinct geographical variation [4–8].

Native joint septic arthritis is usually secondary to hematogenous seeding of joint during transient or persistent bacteraemia [1, 2, 9]. Early treatment is essential before damage to the articular cartilage occurs. This mandates empirical antibiotic therapy without awaiting culture report [1, 2, 4]. Because of wide variation in microbiological spectrum and their sensitivity, a constant antibiotic regimen cannot be designed for all children. Accordingly, this prospective study was conducted to evaluate the microorganisms and their sensitivity pattern in septic arthritis of children who attended our tertiary care centre. Since this centre caters the needs of most of the states of North India, it may be helpful in generating regional data and providing guidelines so as to reduce irrational use of antibiotics and development of antibiotic resistance.

## 2. Materials and Methods

Fifty children of either sex, presenting to the Orthopaedics Department of the institute with septic arthritis of any joint, were included in this two-year prospective study to evaluate the microorganisms and their sensitivity pattern. Children in the age group of 1–12 years presenting with joint pain, fever, restriction of movements and elevated cell counts with antibiotic treatment for not more than 48 hours were included and evaluated. Confirmation of diagnosis was done by (a) at least one of the following radiological or sonographic findings: increased joint space and bony changes or fluid collection on ultrasound scan with (b) purulent fluid aspiration from the joint.

The involved joint was aspirated under sterile precautions in a separate procedure room and 2 cc aspirate was sent for gram stain and culture. We used Robertson Cooked Meat Media to transport the aspirated material for culture. Simultaneously, two blood culture samples were taken and empirical antibiotic therapy in the form of intravenous amoxicillin and clavulanic acid with an aminoglycoside was started, as per the protocol of our department. 

The aspirate was cultured both in aerobic and anaerobic medium following standard protocols. Mackonkey agar, blood agar, and chocolate agar were used for inoculation. Anaerobic culture was done using Anoxomat automated system. The microorganisms were incubated for 48 hours and results were analysed. Sensitivity was tested against the following antimicrobial drugs: cloxacillin, amoxicillin, ceftriaxone, linezolid, and vancomycin. Antibiotic susceptibility tests were done by disc diffusion method of Clinical Laboratory Standards Institute (CLSI). 

The children were treated either with arthrotomy or antibiotic therapy depending on their severity and time of presentation. Definite antibiotic treatment for a period of 6 weeks was given as per the culture report. The children were followed up with clinical and haematological investigations every week for a period of 12 weeks.

## 3. Results

The mean age of presentation was 7.54 years with 70% of children older than five years. Male children outnumbered the female children in the ratio of 2.3 : 1. Sixty-eight percent of children presented within a week, 24% presented in the second week, and 8% presented after two weeks. Lower extremities were involved in 88% of the cases (50% hip, 36% knee, and 2% ankle joint). In the upper extremities, the involvement of shoulder and elbow joint was 10% and 2%, respectively. Most of the children (74%) had moderate presentation (Table 1). 

Twenty-five patients (50%) received amoxicillin, 21 patients (42%) received cloxacillin, and 4 patients (8%) received ceftriaxone as empirical therapy. There was an average decrease in total leukocyte counts from 12000/mm³ at baseline to 8500/mm³ at 12-week followup. Similarly mean ESR and CRP also showed a gradual decline with treatment (Table 2). 

Radiologically, half of the cases (50%) showed positive findings like increased joint space, haziness, and soft tissue changes. Ultrasonography showed evidence of fluid collection in all cases (98%) except one where the findings were equivocal. Gram stain showed gram-positive cocci in 35 samples (70%). Blood culture could not isolate any organism in 32 cases (64%). In 17 cases (34%), it showed *S. aureus* and rest one case (2%) showed Gr. B *Streptococcus*. Aspirated fluid/pus culture yielded better results and 31 cases (62%) had *S. aureus* growth, 3 (6%) had Gr. B *Streptococcus*, and 2 (4%) had *S. pneumoniae*. In 14 cases (28%), it could not isolate the organisms. Based on culture report, antibiotics were changed in 16 cases (32%). Sixty-one percent of the samples were sensitive to cloxacillin, 69% sensitive to amoxicillin, and 83% sensitive to ceftriaxone. None of the organisms were found resistant to vancomycin and linezolid.

## 4. Discussion

Despite extensive studies on acute septic arthritis in childhood, poor outcomes continue to occur. Most important factors determining the outcome of septic arthritis are rapid diagnosis and timely intervention in the form of effective antibiotics and surgical drainage [1, 2, 10]. This study is an attempt to redefine effective empirical therapy and to reinforce the importance of common organisms found in these patients. 

**Figure 2:**
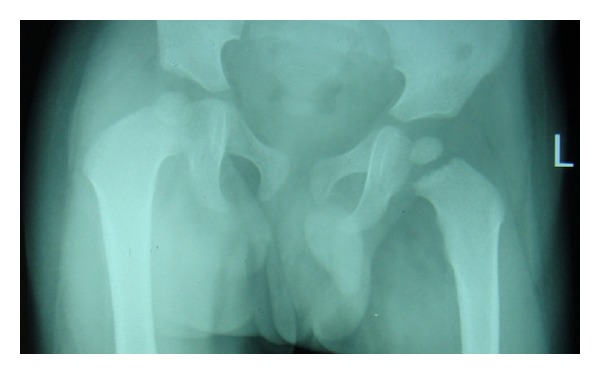
Septic arthritis of left hip showing increased joint space.

Classically, the diagnosis of septic arthritis is made on the basis of clinical examination as the child presents with high grade fever (>38°C) and a painful swollen joint in the absence of history of trauma. Clinical signs may be subtle with irritability, low-grade fever, and limitation of movements. Atypical clinical presentation can also be due to prior antibiotic treatment. The diagnostic tools like radiographs (Figure 2) and ultrasound may aid in diagnosis. Pus on aspiration is almost diagnostic [1–4]. The diagnosis in the present study was primarily clinical which was confirmed by aspiration of the joint. 

The literature has enough evidence in support of ESR and CRP, which can be used as reliable markers in septic arthritis [1, 2, 11, 12]. This study also found a raised ESR and CRP level which persistently declined with antibiotic treatment. As a prognostic marker these two parameters can fairly indicate the severity of septic arthritis.

We agree with Buxton that radiological changes may not be noticed in 50% of the children. However its importance in diagnosis of underlying osteomyelitis cannot be denied [13]. This can alter the management protocol of the disease. Ultrasound is an excellent diagnostic tool that can reliably establish the diagnosis by indicating the presence of fluid in the joint and can guide aspiration. Gordon reported 5% incidence of false negative sonographic scan while diagnosing septic arthritis. Two of these patients presented within 24 hours and in other two the scan was inadequate [14]. Only one child in this study with mild clinical presentation had negative sonographic scan.

The present study showed *S. aureus* as the most common (62%) organism causing septic arthritis in North India, followed by Gr. B *Streptococcus* and *S. pneumoniae*. The observation of Arnold, who reported *H. influenzae* as an important cause of septic arthritis in third world nations, is not true for Indian subcontinent [5]. We could not isolate this organism in any of our patients. This is attributed to the inclusion of *H. influenzae* vaccination protocol in the immunization schedule. Our study is well supported by another study conducted in our institute by Narang et al., who found *Klebsiella pneumoniae* and *S. aureus* as common isolates. But this was a retrospective study conducted in neonates [15]. 

The commonly used empirical therapy in osteoarticular infection is cloxacillin/amoxicillin and clavulanic acid and amikacin combination [1, 2, 4, 16]. Sometimes third generation cephalosporin (commonly ceftriaxone) is started as the initial treatment. However the present study concluded that cloxacillin, amoxicillin, and ceftriaxone were sensitive only in 61%, 69%, and 83% of the cases, respectively. This puts question mark on their efficacy to be used as empirical drugs especially in joint sepsis which may have devastating consequences. Indirectly, this was evident as we needed to change the empirical antibiotics as per culture and sensitivity pattern in 32% cases. However, none of the organisms isolated were resistant to vancomycin and linezolid.

## 5. Conclusion

The most common causative organism of septic arthritisin children is still *S. aureus*. However the common antibiotics (cloxacillin, ceftriaxone) may not be effective as an empirical therapy and due consideration should be given to more effective drugs like vancomycin.

## Figures and Tables

**Table 1:** Severity of disease versus time of presentation.

Severity of disease	Time of presentation		
	<7 d	7 to 14 d	>14 d
Mild	7	0	0
Mod	27	8	2
Severe	0	4	2

**Table 2:** Trend of total leucocyte count, ESR, and CRP with respect to time.

Time scale	Parameters		
	TLC	ESR	CRP
	Mean ± SD	Mean ± SD	Mean ± SD
Baseline	12160 ± 1868	31.6 ± 8.06	2.57 ± 0.48
Two weeks	10518 ± 1101	26.3 ± 5.26	2.04 ± 0.38
Six weeks	9481 ± 740	19.4 ± 4.5	1.57 ± 0.41
Twelve weeks	8470 ± 722	11.7 ± 3.15	0.97 ± 0.33
